# Interspecific variation of warning calls in piranhas: a comparative analysis

**DOI:** 10.1038/srep36127

**Published:** 2016-10-26

**Authors:** Geoffrey Mélotte, Régis Vigouroux, Christian Michel, Eric Parmentier

**Affiliations:** 1 Laboratoire de Morphologie Fonctionnelle et Evolutive, Institut de Chimie, Bât. B6c, Université de Liège, B-4000 Liège, Belgium; 2 HYDRECO Guyane, Laboratoire Environnement de Petit Saut, B.P. 823-97388 Kourou Cedex, French Guiana; 3 Département de Biologie, Ecologie et Evolution, AFFISH Research Center, Université de Liège, Institut de Zoologie, 22 quai Van Beneden, B-4020 Liège, Belgium

## Abstract

Fish sounds are known to be species-specific, possessing unique temporal and spectral features. We have recorded and compared sounds in eight piranha species to evaluate the potential role of acoustic communication as a driving force in clade diversification. All piranha species showed the same kind of sound-producing mechanism: sonic muscles originate on vertebrae and attach to a tendon surrounding the bladder ventrally. Contractions of the sound-producing muscles force swimbladder vibration and dictate the fundamental frequency. It results the calling features of the eight piranha species logically share many common characteristics. In all the species, the calls are harmonic sounds composed of multiple continuous cycles. However, the sounds of *Serrasalmus elongatus* (higher number of cycles and high fundamental frequency) and *S. manueli* (long cycle periods and low fundamental frequency) are clearly distinguishable from the other species. The sonic mechanism being largely conserved throughout piranha evolution, acoustic communication can hardly be considered as the main driving force in the diversification process. However, sounds of some species are clearly distinguishable despite the short space for variations supporting the need for specific communication. Behavioural studies are needed to clearly understand the eventual role of the calls during spawning events.

Acoustic signals may amongst other things convey information relative to species identity^1^. Several examples demonstrating divergence in acoustic signals between closely-related species can be found in different sound-producing taxa such as arthropods^2^,^3^, anurans^4^,^5^,^6^, birds^7^,^8^ and mammals^9^,^10^. These differences would correspond to adaptations to different constraints and could help interspecies discrimination, preventing interbreeding.

Among the vertebrates, fishes have developed the greatest diversity of sound-producing mechanisms^11^,^12^ and are able to produce sounds in various behavioural contexts^12^,^13^,^14^,^15^,^16^,^17^,^18^. Few studies have discussed call diversity and evolution in closely related fish species. In some Gobiidae, Batrachoididae and Pomacentridae, characteristics of the acoustic signals and similarities between the sound-producing mechanisms support affinities between different groups of species^16^,^19^,^20^,^21^. However, calls of the different taxa provide different kinds of information and are not necessarily produced in the same behavioural context. For example, the differences at the level of pulse duration and dominant frequency between clownfish species are mainly due to differences in the species size^22^,^23^,^24^. The high overlap in call features suggests that sounds are not the main driving force in the diversification process of this clade. However, calls in clownfish are not used during courtship periods but only during agonistic interactions^15^ meaning there is no sexual selection based on call features. In the Mediterranean gobies, call diversification is more important since related species show less overlap of their call features and have even developed different kinds of calls, i.e., pulsatile, tonal and complex sounds^20^,^21^. Sounds are used in reproductive contexts meaning the selective forces driving the diversification are more important. This higher diversity in sounds would support the role of acoustic communication in the species’ evolution.

Piranhas are neotropical freshwater fishes belonging to Serrasalmidae^25^. Recent investigations support the monophyly of this family, which is currently divided into three major clades having vernacular names: the “pacu”, the “*Myleus*” and the “true piranhas”^26^,^27^,^28^. Within the Serrasalmidae, sound production has been studied in a few piranha species while the fish were hand-held^29^,^30^,^31^ but some authors do not provide the species name, making their studies less useful. In the red bellied piranha (*Pygocentrus nattereri*), Millot *et al*.^32^ identified three types of sounds and associated each one with a particular behaviour. One sound is characterized by a high frequency (1700 Hz) and is caused by a rapid closure of the jaws. This sound is produced when the fish attempts to bite a conspecific or a prey. Another sound contains only one pulse. Its fundamental frequency is about 43 Hz and this sound is emitted in a context of food competition. The last type of sound consists of many pulses/cycles and its fundamental frequency is about 120 Hz. In natural conditions, it would correspond to a warning/agonistic signal during a confrontation between two individuals^32^. The second and third kinds of sound are the result of the rapid contraction of a pair of extrinsic muscles vibrating the cranial sac of the swimbladder^29^,^30^,^31^. Sound-producing muscles are on each side of the swimbladder. They originate at the broad proximal base of the second rib and are on a large tendon ventrally surrounding the anterior part of the swimbladder^33^. Kastberger^30^ showed that the contraction rate of drumming muscles in piranhas sets the fundamental frequency of the acoustic signals. The observation that the swimbladder of *P. nattereri* is a highly damped structure confirms nicely the study of Kastberger because it shows the oscillation frequency only depends on the contraction rate of sonic muscles and not on the swimbladder resonant frequency^32^.

The present study focuses on the acoustic features in eight Serrasalmidae species belonging to the flesh-eating piranhas^27^,^28^. These species were *Serrasalmus elongatus* Kner, 1858; *Serrasalmus marginatus* Valenciennes, 1837; *Serrasalmus compressus* Jégu, Léão & Santos, 1991; *Serrasalmus manueli* (Fernández-Yépez & Ramírez, 1967); *Serrasalmus spilopleura* Kner, 1858; *Serrasalmus rhombeus* (Linnaeus, 1766); *Serrasalmus eigenmanni* Norman, 1929 and *Pygocentrus nattereri* Kner, 1858. The first aim of this study was to describe the warning signals produced by these closely related species and to compare their acoustic features. The second objective was to evaluate, by means of the acoustic properties of the different species, the potential role of acoustic communication as a driving force in the diversification of piranhas.

## Results

### Structural properties of acoustic signals

**Figure 1:**
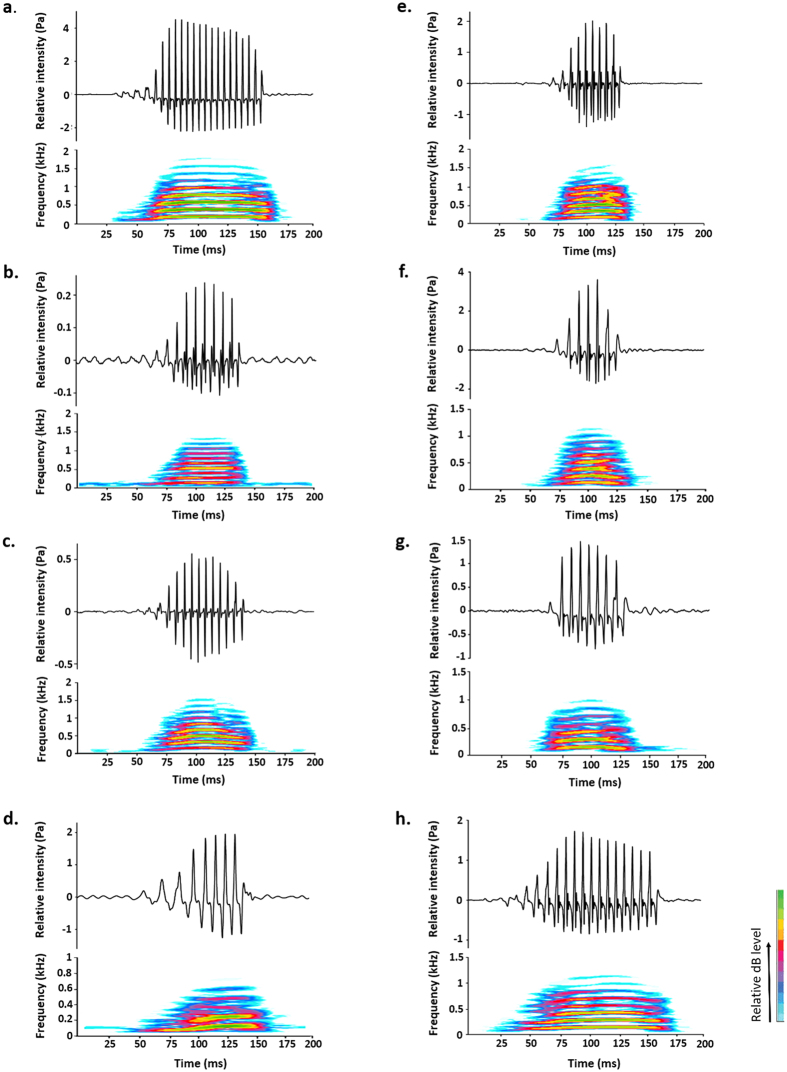
Waveform (above) and spectrogram (below) of the sounds produced by (**a**) *Serrasalmus elongatus*; (**b**) *Serrasalmus marginatus*; (**c**) *Serrasalmus compressus*; (**d**) *Serrasalmus manueli*; (**e**) *Serrasalmus spilopleura*; **(f**) *Serrasalmus rhombeus*; (**g**) *Serrasalmus eigenmanni*; (**h**) *Pygocentrus nattereri*. Sounds were recorded at 44.1 kHz.

The eight species showed common characteristics in their acoustic signals. All sounds consisted of multiple continuous cycles. In all piranha species, sound duration was highly positively correlated to the number of cycles. The first two to five cycles in the sound had a lower amplitude than the successive ones (Fig. 1). In all species, the first cycle period was significantly longer than the mean cycle period of the following cycles (Wilcoxon signed-rank test; *P *< 0.001 for all species). Moreover, in each species, the cycle period progressively decreased during the sounds’ emission (see Supplementary Fig. S1). The acoustic signals possessed a fundamental frequency between 100 and 180 Hz and harmonics (Table 1).

### Interspecific variation of acoustic signals

For all the measured acoustic variables, at least one species differed significantly from the others (Kruskal-Wallis test, *P *< 0.001 for each acoustic property; see Supplementary Table S1).

*Serrasalmus elongatus*, *S. rhombeus* and *P. nattereri* were the three species that significantly differed from the others for sound duration (Dunn’s multiple comparison test, *P *< 0.001; see Supplementary Table S1). This difference was clearly related to the number of cycles. *Serrasalmus elongatus* produced the highest number of cycles and possessed thus the longest calls whereas *S. rhombeus* produced the lowest number of cycles and thus the shortest calls (Table 1). *Pygocentrus nattereri* was in an intermediate position between these two species. The five other species cannot be statistically separated on the basis of this temporal feature.

*Serrasalmus manueli* (Table 1) had the longest cycle period and the lowest fundamental frequency (mean value around 104 Hz; Dunn’s multiple comparison test, *P* < 0.001; see Supplementary Table S1). *Serrasalmus elongatus* had the shortest cycle period and highest fundamental frequency (mean value around 172 Hz; Dunn’s multiple comparison test, *P *< 0.001; Table 1). The fundamental frequencies of the other species were intermediate between these two species (*S. manueli* and *S. elongatus*) but separated into two different groups: the first group comprises *S. rhombeus* and *S. eigenmanni* (mean value around 124 Hz) and the second one comprises *S. marginatus*, *S. compressus*, *S. spilopleura* and *P. nattereri* (mean value around 147 Hz; Table 1).

The number of cycles was highly correlated to the sound duration (Pearson correlation coefficient: r = 0.918, *P *< 0.001). This was also the case between the cycle period and the fundamental frequency (Pearson correlation coefficient: r = −0.955, *P *< 0.001). Consequently, the variables “number of cycles” and “cycle period” were removed from statistical analyses involving principal component analysis (PCA) and discriminant function analysis (DFA).

**Figure 2:**
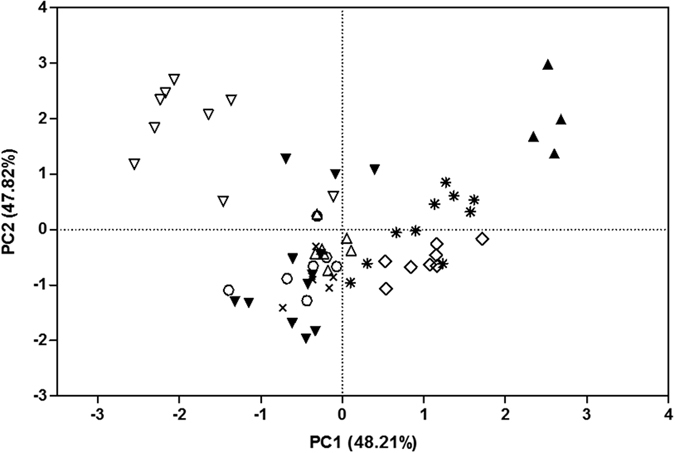
Scatterplot of principal component (PC) 1 versus PC2, performed with individual mean values of the three acoustic properties. Legend: *Serrasalmus elongatus* ▽, *Serrasalmus marginatus* ×, *Serrasalmus compressus* △, *Serrasalmus manueli* ▲, *Serrasalmus spilopleura* ○, *Serrasalmus rhombeus* ■, *Serrasalmus eigenmanni* ■ and *Pygocentrus nattereri* ▼.

The first two principal components of the PCA explained cumulatively 96.03% of the variation, with PC1 and PC2 explaining, respectively, 48.21% and 47.82% of the variation. Fundamental frequency mostly contributed to the first principal component, whereas temporal variables (“sound duration” and “first cycle period”) were principally associated with the second principal component (see Supplementary Table S2). The plot of the species along the first two principal components showed that several species can be distinguished from the others (Fig. 2). The species characterised by a high fundamental frequency, such as *S. elongatus* and, to a lesser extent, *S. marginatus*, *S. compressus*, *S. spilopleura*, and *P. nattereri*, are found on the left part of the diagram, whereas species with a low fundamental frequency, such as *S. manueli*, *S. rhombeus*, and *S. eigenmanni*, are on the right. Along axis 2, *S. elongatus* and *S. manueli* are segregated from the other species. *Serrasalmus elongatus* and *S. manueli* are characterised by a long sound duration and a long first cycle period, respectively (Table 1).

**Figure 3:**
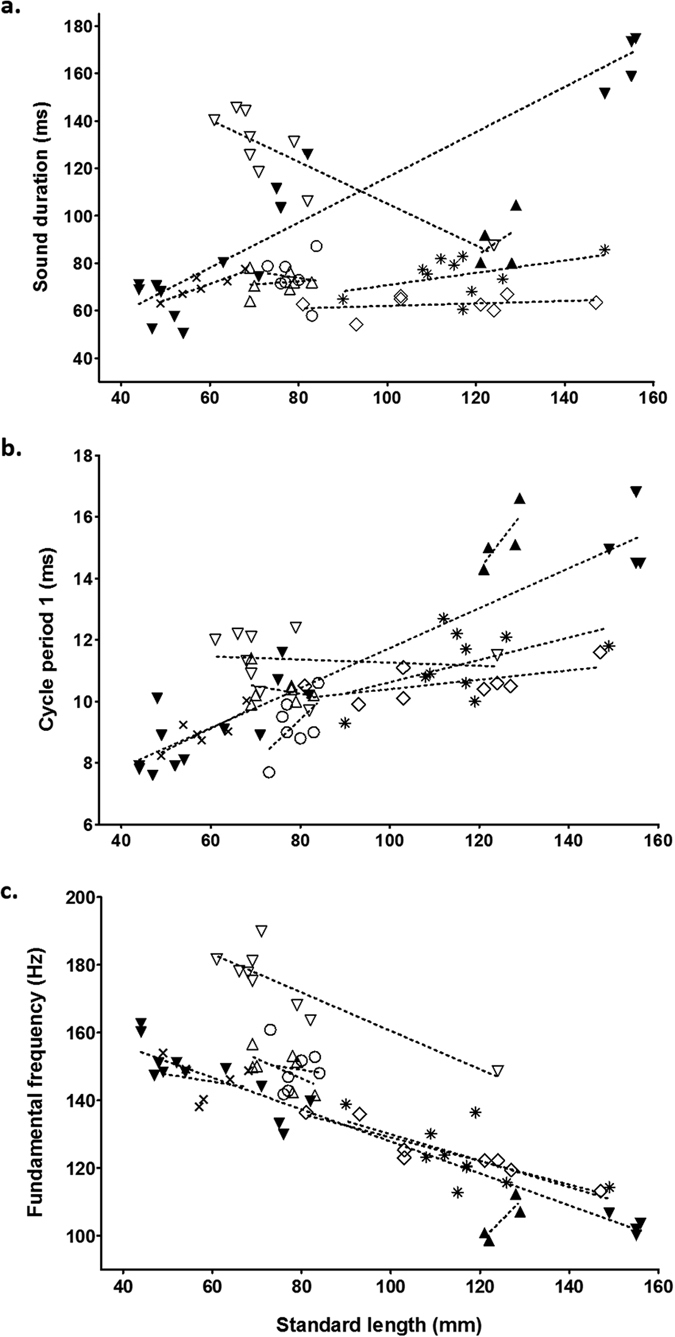
Influence of fish size on **(a)** sound duration, **(b)** cycle period 1, and **(c)** fundamental frequency in 8 piranha species. Note that some data concerning *P. nattereri* have been added from Millot and Parmentier^48^. Legend: *Serrasalmus elongatus* ▽, *Serrasalmus marginatus* ×*, Serrasalmus compressus* △, *Serrasalmus manueli* ▲, *Serrasalmus spilopleura* ○, *Serrasalmus rhombeus* *, *Serrasalmus eigenmanni* ■ and *Pygocentrus nattereri* ▼.

The three variables (sound duration, first cycle period, fundamental frequency), being associated with PC1 and PC2, were regressed on fish body size for each species (Table 2, Fig. 3). The regression lines were not all significant but some tendencies were highlighted according to the species (Table 2). The sound duration is positively related to an increase in fish size except in *S. elongatus* and *S. spilopleura* which were characterised by a negative relationship (Table 2, Fig. 3a). It is worth mentioning the weak relationship in *S. spilopleura* and *S. compressus* could be due to the small range in size. Additional bigger specimens are probably needed to reinforce the shown data.

As for the sound duration, the first cycle period seems to increase with fish body size, with the exception of *S. elongatus* and *S. compressus* (Table 2, Fig. 3b). Once again, the results concerning *S. compressus* are subject to caution.

Figure 3c reveals that fundamental frequency decreased with fish size although the size range in *S. manueli* was too small to allow a conclusion.

The discriminant function analysis (DFA) highlighted the predominant roles of fundamental frequency on the DF1 and first cycle period on the DF2 to discriminate the eight piranha species (see Supplementary Table S3). Sounds were associated with the correct species, with an overall correct classification rate of 53.6%. The correct classification rates vary according to the species: *Serrasalmus elongatus* (80%), *Serrasalmus manueli* (90.1%) and, to a lesser extent, *Serrasalmus rhombeus* (60%) were largely correctly classified. However, the accuracy of categorisation was only 34% in *Serrasalmus marginatus,* 56.4% in *Serrasalmus compressus,* 26.9% in *Serrasalmus spilopleura,* 50.6% in *Serrasalmus eigenmanni* and 32.2% in *Pygocentrus nattereri*, indicating that the sound parameters of these five latter species are more variable than in the three former.

**Figure 4:**
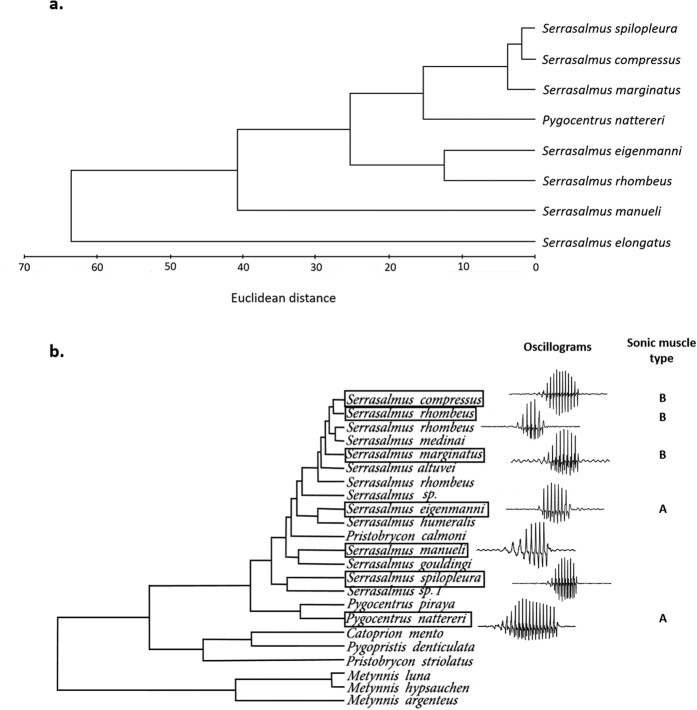
Comparison between the acoustic affinities within the piranha species investigated and their phylogenetic affinities. (**a**) UPGMA phenogram resulting from the cluster analysis based on the means of the acoustic properties (UPGMA, Euclidean distances). (**b**) Molecular phylogeny of the “true piranhas” clade extracted and adapted from Thompson *et al*.^28^. A box is drawn around the species investigated in our study. A representative waveform of the species’ call and the sonic muscles type are shown for each species analysed. A, sonic muscles extending from the first to the third ribs; B, sonic muscles extending from the first to the fourth ribs.

The UPGMA phenogram illustrated the degree of acoustic signal similarity between species (Fig. 4a). It revealed that *S. elongatus* has a well separate position with respect to the other species, which branch off into *S. manueli* and a cluster composed of all the other species. This cluster branches off into two main subclusters: one formed by *S. marginatus*, *S. compressus*, *S. spilopleura*, and *P. nattereri* and the other composed of *S. rhombeus* and *S. eigenmanni*. In the former subcluster, *P. nattereri* is separated from a group composed of the three other species, in which *S. compressus* and *S. spilopleura* seem to be sister species.

### Morphology of the sound-producing apparatus

**Figure 5:**
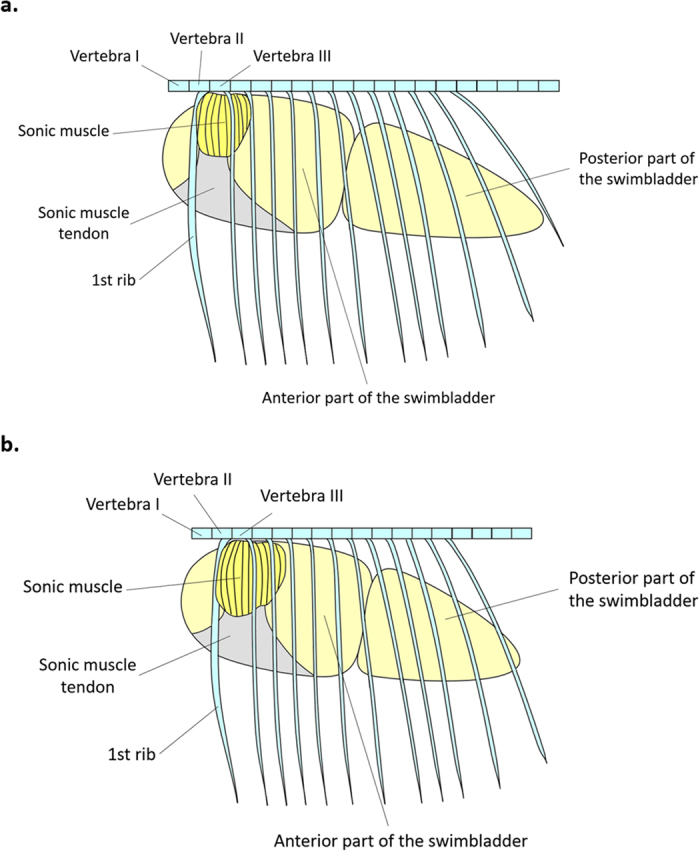
Schematic left lateral view of the sound-producing apparatus in **(a)** *Pygocentrus nattereri* and **(b)** *Serrasalmus compressus*. Note that the same sonic mechanism as in **(a)** is found in *S. eigenmanni*, whereas *S. elongatus*, *S. marginatus* and *S. rhombeus* possess the same mechanism as in **(b)**.

The six investigated piranha species possessed sonic muscles associated to the anterior sac of the swimbladder. A morphological difference occurred between a group composed of *S. eigenmanni* and *P. nattereri*, and another group comprising the other *Serrasalmus* species. *Serrasalmus eigenmanni* and *P. nattereri* possess sonic muscles extending from the first to the third ribs, whereas the other *Serrasalmus* species have sonic muscles extending from the first to the fourth ribs (Fig. 5). Morphological measurements revealed no species-specific features that could be associated with sound characteristics of the different species (see Supplementary Table S4).

## Discussion

All species produced calls when fishes were hand-held. Moreover, to the best of our knowledge, this was the first time that sounds produced by *S. marginatus*, *S. compressus*, *S. manueli* and *S. eigenmanni* have been recorded.

The morphology of the sound-producing apparatus is similar in the eight piranha species and corresponds to the descriptions already given for this clade^29^,^30^,^31^,^32^. Having the same kind of sound-producing mechanism, it is logical that the calling features share common characteristics. One example would be harmonic calls, made of trains of cycles having a duration of *ca* 80 ms and a mean fundamental frequency between 100 and 200 Hz. In all the species, the first cycle is longer and of weaker amplitude than the consecutive cycles. According to Fine *et al*.^34^, this is also observed in another fish making sounds with swimbladder-related sonic muscles, the toadfish. The interpretation of the authors was that the release and the uptake of calcium from the sarcoplasmic reticulum would be lower during the first contractions^34^.

Although there is an overlap in acoustic parameters, as shown in the PCA, interspecific comparisons revealed that, for each acoustic property, at least one species differed significantly from the others. *Serrasalmus elongatus* is clearly distinguishable by having a higher number of cycles and frequency than the other seven species, whereas *S. manueli* is characterised by the longest cycle periods and thus the lowest fundamental frequency (Table 1). Significant differences are also found in some features among the sounds of the other six species, but they do not allow clear acoustic distinction. These results were confirmed by the discriminant function analysis which revealed that only the sounds of *S. elongatus, S. manueli* and, to a lesser extent, *S. rhombeus* were correctly assigned.

Due to the relative simplicity of vocal mechanisms, fish typically lack the ability to produce complex calls^35^,^36^, meaning they do not have many ways to modify their calls. Variations in sounds can be mainly achieved by modifications in either temporal patterning or frequency^19^. The sound duration (including the number of cycles) can be explained by neural mechanisms that command the number of muscle contractions^37^,^38^,^39^. The mean differences in the main frequencies (including cycle periods) can be explained by both the muscle contraction rates and the size of the swimbladder^12^,^40^. Statistical analyses support the distinctiveness in most of the piranha sounds. However, the comparisons between species show that some of these differences are really small (±70 Hz in main frequency, ±3 ms in the cycle period), suggesting play back experiments should be done to test the species ability to discriminate the specific calls; certainly human ear perception does not allow for distinguishing between piranha species except for the case of *S. elongatus* and *S. manueli*.

As an alternative to making modifications in the properties of a kind of sound, some phylogenetically-related species have also developed different mechanisms. In the butterflyfishes, for example, different kinds of sounds at frequencies of <1 to >1000 Hz can be produced by different, unrelated, mechanisms such as head bob or tail slap^41^. Several catfish families are able to produce sounds by means of two sonic organs: pectoral spines for stridulation and swimbladder drumming muscles^42^,^43^,^44^. It was shown in *P. nattereri* that different kinds of sounds can be made with distinct mechanisms^32^. This diversification supports the idea that acoustic communication could be important in *P. nattereri* but this ability is not known in other serrasalmid species.

Fish body size (Table 1) seems to be closely related to some acoustic features (sound duration, first cycle period and fundamental frequency). The relationship between size and dominant frequency (Table 2, Fig. 3c) is well known in fishes^40^ and in many other taxa^45^,^46^. The exceptional finding in *S. manueli* (Fig. 3c) is probably due to the small range of size and deserves further observations. Although the sound-producing mechanism is completely different^47^, analogous observation has been realised in the clownfish clade, in which size predicted the dominant frequency across 14 species. In clownfish, all the studied species were on the same slope supporting the hypothesis that the mechanism is highly conservative in this taxa^22^. As in piranhas, differentiations in the calls of the clownfish species can be made by changing the number of pulses or varying the period. In clownfish, speciation could be related to modifications of adult size; differences in the calls being a by-product of these variations. For example, the calling frequency of *Premnas biaculeatus* (110 mm) is around 400 Hz whereas it is between 700 and 900 Hz in the sister clade comprising *Amphiprion ocellaris* and *Amphiprion percula* (40–60 mm)^22^. Variations related to size are also mathematically found in piranhas. However, contrary to the damselfish, the effect is relatively small. In *P. nattereri*, there is a difference of 50 Hz between fish of 45 mm and 150 mm^48^. In piranhas, the dominant frequency is more dependent on the contraction rate than fish size, meaning it is difficult to argue that piranhas use size as a route to diversification. If it turned out this actually was the case, it is probably related to other causes than the production of sounds.

Contrary to clownfish, sound production in piranhas is more dependent on physiological features such as the contraction of the high-speed muscles. In clownfish, the frequency is related to size whereas it is related to size and the muscle physiology in piranhas. As a result, all the species are not on the same slope in the frequency-size relationship. This observation is also shown for other features such as the first period, the number of cycles and the sound duration.

The current rate of diversification of piranhas is the highest reported to date in serrasalmids, and sympatric speciation may play a significant role in increasing diversity in the Amazon^49^. The history of piranhas has been quite complex, resulting from a mixed occurrence of vicariant and dispersal events^49^. Most of the eight piranha species in our study have a large distribution across South American river systems^25^ and each species lives in sympatry with at least another one. Nevertheless, the different species produce the same types of sound with common acoustic features despite sympatry of many of them. A hypothesis is that sounds are used during agonistic interactions towards individuals of different species.

The phenogram illustrating the acoustic affinities among the eight piranha species (Fig. 4a) does not show any similarity with the last phylogenetic relationships of the “true piranhas” clade^28^ (Fig. 4b). Species showing the strongest differences in acoustic features (*S. manueli*) do not occupy a particular position in the tree. Unfortunately, *Serrasalmus elongatus* that has the most peculiar sound features, was not incorporated in the phylogenetic studies found in the literature. *Serrasalmus manueli* is not geographically isolated^25^ and its sounds could be a way to discriminate from other species but this conclusion cannot be applied to all the taxa in the same situation. Contrary to what we found in piranhas, previous studies suggest that, in Mediterranean gobies^20^ and in *Dascyllus* species^16^, there is congruence between the acoustic-based affinities among species and those obtained by means of genetic data. Although our results cannot reveal the role features of sounds play in species identification (except in *S. elongatus* and *S. manueli*), the complexity of the sound-producing mechanism indicates that acoustic communication must be important within this fish group. Future studies are required to find out more about the eventual role of sounds in the success of this clade.

Our results suggest that acoustic communication cannot be considered as the main driving force behind the diversification of piranha species. However, some species found the way to be clearly distinguishable despite the short space for variations supporting the need for distinct communication message. Because the sound producing mechanism seems largely conserved, it seems fish have mainly utilized neural mechanisms to expand the repertoire. Further studies are required to investigate the eventual role sounds play during spawning periods.

## Methods

### Fish collection

All the Serrasalmidae species except *S. rhombeus* and *S. eigenmanni* were purchased in the aquarium trade. They were housed in freshwater aquaria at 26 ± 1 °C and were maintained on a 12 h light/dark cycle. The tanks were equipped with external filters, internal heaters and bubblers for the oxygenation of the water. Fish were fed with mussels three times a week. *Serrasalmus rhombeus* and *S. eigenmanni* were caught by means of gill nets in March 2015 during a field mission on the Tampok and Waki rivers in French Guiana (Guiana Amazonian Park).

### Sound collection and analysis

Sixty-three specimens belonging to the eight pre-identified species were audio-recorded. Several species of the present study were recorded for the first time. For all the species except *S. rhombeus* and *S. eigenmanni*, sounds were recorded in aquaria using a hydrophone (HTI-96-MIN Series; High Tech, MS, USA; sensitivity: -164.4 dB re 1 V/μPa) connected to a portable stereo recorder (Tascam DR – 05). During the recordings, temperature was 26 ± 1 °C while lighting, external filters and bubblers were turned off to reduce background noise. All fish were recorded whilst hand-held because piranhas are known to emit warning calls in such situations^29^,^30^,^31^,^32^. To standardize the recordings, fishes were recorded in the same tank (1.5 × 0.5 × 0.4 m) at a distance of approximately 5 cm from the hydrophone. This distance also enables fish to remain within the attenuation distance described by Akamatsu *et al*.^50^. *Serrasalmus rhombeus* and *S. eigenmanni* were both recorded in the field in a tank without electrical appliances. The recording material and the recording conditions were otherwise the same as previously described for the other species.

**Figure 6:**
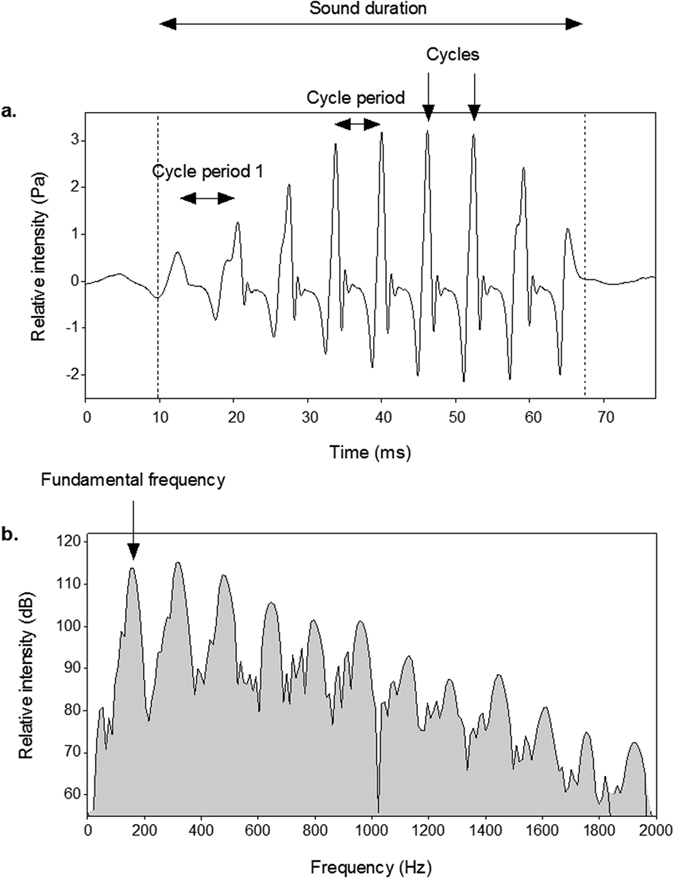
Diagram illustrating the five measured acoustic variables. **(a)** Oscillogram used to measure temporal features of the sound: sound duration (ms), number of cycles in the sound, first cycle period (measured as the peak-to-peak interval between the first two cycles in the sound, ms); cycle period (measured as the average peak-to-peak interval between consecutive cycles in the entire sound except the first two cycles, ms). **(b)** Power spectrum used to determine fundamental frequency (frequency which corresponds to the first harmonic in the power spectrum, Hz), marked with an arrow. The other peaks correspond to the harmonics.

Sounds were digitised at 44.1 kHz (16 bit-resolution) and analysed using Avisoft SAS-Lab Pro 5.2 software. All recordings were bandpass filtered (band: 50–2000 Hz) to avoid frequency distortion due to the resonance of the tank^50^. Temporal features were measured from oscillograms, and frequency parameters were obtained from power spectra. A spectrogram of the sounds produced by each species was obtained to visualize the frequency components (FFT length: 256 points, frame size: 100%, window: flat top, time overlap: 98.43%). The sound parameters measured were (Fig. 6): sound duration (millisecond, ms); number of cycles in a sound; first cycle period (measured as the peak-to-peak interval between the first two cycles in the sound, ms); cycle period (measured as the average peak-to-peak interval between consecutive cycles in the entire sound except the first two cycles, ms); and fundamental frequency (Hz), which represents the first harmonic in the power spectrum. There was no inter-cycles interval in the analysed sounds; therefore, cycle length was not measured because it represents the same measure as cycle period.

### Morphological study

In order to observe and to compare the sound-producing apparatus in piranhas, fifteen specimens belonging to six different piranha species were dissected. The investigated species were: *S. elongatus*, *S. marginatus*, *S. compressus*, *S. rhombeus*, *S. eigenmanni* and *P. nattereri*. Specimens belonging to *S. manueli* and *S. spilopleura* were not dissected because they were maintained alive for further experiments not related to this study. The individuals were euthanized with tricaine methanesulphonate MS-222 and fixed in 7% formalin for approximately two weeks before being transferred to 70% ethanol for storage. Specimens were dissected at the level of the anterior sac of the swimbladder and examined with a Wild M10 (Leica) binocular microscope. In order to highlight potential gross morphological differences among species, dimensions of the anterior sac of the swimbladder and of the sonic muscle were measured using a caliper.

### Ethical statement

All procedures and all methods were approved by the ethical commission of the University of Liège (ethics case 1532). All experiments were performed in accordance with the relevant guidelines and regulations.

### Statistical analysis

Descriptive statistics were calculated for each temporal and frequency variable of the sounds produced by fishes. A Wilcoxon signed-rank test was performed for each species to compare the first cycle period in the sound to the mean cycle period of the following cycles. The acoustic variables were then tested for the assumption of normality (Shapiro-Wilk test), and analysed using a non-parametric Kruskal-Wallis test followed by subsequent Dunn’s multiple comparison test for pairwise comparisons between species. The data used for the comparison of acoustic properties among species were all the recorded sounds produced by each individual.

To determine which sound properties were useful for the subsequent analyses, the individual means of the five acoustic properties were first tested for correlation using the Pearson correlation. Then, to compare overall similarities between the species, principal component analysis (PCA) was run on the individual means of the acoustic variables proven to be independent. The means of the sonic properties which mostly contributed to the PCA components were regressed against fish body size to test the prospective dependence of the acoustic variables on this independent factor.

A discriminant function analysis (DFA) was also carried out on the sound properties data. This analysis aims to determine which variables differentiate the species. It also permits an assessment of how well sounds can be assigned to the correct species of piranhas.

Finally, a cluster analysis (unweighted pair-group method using arithmetic mean, UPGMA, Euclidean distances) was performed on the means of each acoustic variables for each species. The resulting phenogram illustrated the similarities in call parameters among species.

Statistical analyses were performed with Statistica 12 and GraphPad Prism 5.0. Results are presented as means ± SD. Significance level was determined at *P *< 0.05.

## Additional Information

**How to cite this article**: Mélotte, G. *et al*. Interspecific variation of warning calls in piranhas: a comparative analysis. *Sci. Rep.* **6**, 36127; doi: 10.1038/srep36127 (2016).

**Publisher’s note:** Springer Nature remains neutral with regard to jurisdictional claims in published maps and institutional affiliations.

## Supplementary Material

Supplementary Information

## Figures and Tables

**Table 1:** Mean values and standard deviations of the standard length and the five acoustic variables for the eight species.

Species (N)	n	SL (mm)	Sound duration (ms)	Number of cycles per sound	Cycle period 1 (ms)	Cycle period (ms)	Fundamental frequency (Hz)
*Serrasalmus elongatus* (9)	461	77 ± 19	123.7 ± 29.7	18.6 ± 5.1	11.4 ± 2.1	5.9 ± 0.5	172 ± 14
*Serrasalmus marginatus* (6)	306	58 ± 7	70.5 ± 13.9	10.1 ± 2.0	9.1 ± 1.0	6.9 ± 0.4	146 ± 8
*Serrasalmus compressus* (7)	291	75 ± 6	71.8 ± 12.0	10.0 ± 1.5	10.4 ± 1.7	6.6 ± 0.4	149 ± 8
*Serrasalmus manueli* (4)	121	125 ± 4	89.8 ± 14.8	8.3 ± 1.3	15.2 ± 2.1	8.6 ± 0.6	104 ± 12
*Serrasalmus spilopleura* (7)	305	79 ± 4	73.1 ± 17.8	10.1 ± 2.3	9.2 ± 1.5	6.2 ± 0.4	149 ± 12
*Serrasalmus rhombeus* (8)	400	112 ± 21	62.6 ± 8.8	7.2 ± 1.1	10.6 ± 1.0	7.7 ± 0.6	125 ± 9
*Serrasalmus eigenmanni* (10)	500	116 ± 15	74.9 ± 13.7	8.4 ± 1.4	11.2 ± 1.5	7.9 ± 0.6	124 ± 10
*Pygocentrus nattereri* (12)	205	59 ± 14	86.4 ± 27.6	11.7 ± 3.0	9.5 ± 1.7	6.8 ± 0.6	144 ± 12

N, number of recorded individuals per species; n, number of analysed sounds; SL, standard length.

**Table 2:** Results of the linear regressions of fish size against the three acoustic variables.

Species	Sound duration (ms)			Cycle period 1 (ms)			Fundamental frequency (Hz)		
	Equation	R^2^	*P*-value	Equation	R^2^	*P*-value	Equation	R^2^	*P*-value
*S. elongatus*	*y *= −0.882*x *+ 193.30	0.757	**0.002**	*y *= −0.005*x *+ 11.76	0.011	0.792	*y *= −0.567*x *+ 217.1	0.779	**0.002**
*S. marginatus*	*y *= 0.675*x *+ 30.70	0.810	**0.014**	*y *= 0.071*x *+ 4.875	0.656	0.051	*y *= −0.208*x *+ 158	0.057	0.648
*S. compressus*	*y *= 0.121*x *+ 62.64	0.022	0.753	*y *= −0.025*x *+ 12.25	0.082	0.535	*y *= −0.584*x *+ 193.1	0.366	0.150
*S. manueli*	*y *= 1.220*x* − 63.27	0.185	0.570	*y *= 0.190*x* − 8.50	0.642	0.199	*y *= 1.318*x* − 59.98	0.752	0.133
*S. spilopleura*	*y *= −0.270*x *+ 95.35	0.014	0.800	*y *= 0.138*x* − 1.637	0.356	0.158	*y *= −0.202*x *+ 165.1	0.015	0.794
*S. rhombeus*	*y *= 0.053*x *+ 65.75	0.076	0.509	*y *= 0.015*x *+ 8.874	0.358	0.117	*y *= −0.348*x *+ 163.8	0.874	**0.0007**
*S. eigenmanni*	*y *= 0.259*x *+ 44.86	0.220	0.171	*y *= 0.036*x *+ 6.993	0.256	0.136	*y *= −0.390*x *+ 168.9	0.422	**0.042**
*P. nattereri*	*y *= 0.953*x *+ 20.92	0.911	**<0.0001**	*y *= 0.065*x *+ 5.245	0.912	**<0.0001**	*y *= −0.472*x *+ 175	0.953	**<0.0001**

R^2^, coefficient of determination; *P*-value in bold are significant.
