# Supplementary material for: Interspecific variation of warning calls in piranhas: a comparative analysis
Source: Sci Rep. 2016 Oct 26;6:36127. doi: 10.1038/srep36127 (PMC5080574; doi:10.1038/srep36127)
Supplement: Supplementary Information [file srep36127-s1.pdf]

Interspecific variation of warning calls in piranhas: a comparative analysis

Geoffrey Mélotte[1], Régis Vigouroux[2], Christian Michel[3] and Eric Parmentier[1,*]

[1] *Laboratoire de Morphologie Fonctionnelle et Evolutive, Institut de Chimie, Bât. B6c, Université de Liège, B-4000 Liège, Belgium*
[2] *HYDRECO Guyane, Laboratoire Environnement de Petit Saut, B.P. 823 - 97388 Kourou Cedex, French Guiana*
[3] *Département de Biologie, Ecologie et Evolution, AFFISH Research Center, Université de Liège, Institut de Zoologie, 22 quai Van Beneden, B - 4020 Liège, Belgium*

[*]Author for correspondence (E.Parmentier@ulg.ac.be)

SUPPLEMENTARY INFORMATION

Table S1. Comparisons of the acoustic properties among the eight piranha species.

	Acoustic properties	*P. nattereri*	*S. elongatus*	*S. marginatus*	*S. compressus*	*S. manueli*	*S. spilopleura*	*S. rhombeus*
S. elongatus	SD (ms)	< 0.0001	–	–	–	–	–	–
	NC	< 0.0001	–	–	–	–	–	–
	CP1 (ms)	< 0.0001	–	–	–	–	–	–
	CP (ms)	< 0.0001	–	–	–	–	–	–
	FF (Hz)	< 0.0001	–	–	–	–	–	–
S. marginatus	SD (ms)	< 0.0001	< 0.0001	–	–	–	–	–
	NC	0.001	< 0.0001	–	–	–	–	–
	CP1 (ms)	0.0032	< 0.0001	–	–	–	–	–
	CP (ms)	NS	< 0.0001	–	–	–	–	–
	FF (Hz)	NS	< 0.0001	–	–	–	–	–
S. compressus	SD (ms)	< 0.0001	< 0.0001	NS	–	–	–	–
	NC	0.0034	< 0.0001	NS	–	–	–	–
	CP1 (ms)	< 0.0001	< 0.0001	< 0.0001	–	–	–	–
	CP (ms)	NS	< 0.0001	0.0081	–	–	–	–
	FF (Hz)	NS	< 0.0001	NS	–	–	–	–
S. manueli	SD (ms)	0.0058	< 0.0001	< 0.0001	< 0.0001	–	–	–
	NC	< 0.0001	< 0.0001	< 0.0001	< 0.0001	–	–	–
	CP1 (ms)	< 0.0001	< 0.0001	< 0.0001	< 0.0001	–	–	–
	CP (ms)	< 0.0001	< 0.0001	< 0.0001	< 0.0001	–	–	–
	FF (Hz)	< 0.0001	< 0.0001	< 0.0001	< 0.0001	–	–	–

S. spilopleura	SD (ms)	< 0.0001	< 0.0001	NS	NS	< 0.0001	–	–
	NC	< 0.0001	< 0.0001	NS	NS	< 0.0001	–	–
	CP1 (ms)	NS	< 0.0001	NS	< 0.0001	< 0.0001	–	–
	CP (ms)	< 0.0001	0.0013	< 0.0001	< 0.0001	< 0.0001	–	–
	FF (Hz)	NS	< 0.0001	NS	NS	< 0.0001	–	–
S. rhombeus	SD (ms)	< 0.0001	< 0.0001	< 0.0001	< 0.0001	< 0.0001	< 0.0001	–
	NC	< 0.0001	< 0.0001	< 0.0001	< 0.0001	< 0.0001	< 0.0001	–
	CP1 (ms)	< 0.0001	0.0004	< 0.0001	NS	< 0.0001	< 0.0001	–
	CP (ms)	< 0.0001	< 0.0001	< 0.0001	< 0.0001	< 0.0001	< 0.0001	–
	FF (Hz)	< 0.0001	< 0.0001	< 0.0001	< 0.0001	< 0.0001	< 0.0001	–
S. eigenmanni	SD (ms)	0.0130	< 0.0001	0.0017	NS	< 0.0001	NS	< 0.0001
	NC	< 0.0001	< 0.0001	< 0.0001	< 0.0001	NS	< 0.0001	< 0.0001
	CP1 (ms)	< 0.0001	NS	< 0.0001	< 0.0001	< 0.0001	< 0.0001	0.0003
	CP (ms)	< 0.0001	< 0.0001	< 0.0001	< 0.0001	< 0.0001	< 0.0001	NS
	FF (Hz)	< 0.0001	< 0.0001	< 0.0001	< 0.0001	< 0.0001	< 0.0001	NS

NS, Non-Significant differences; SD, Sound Duration; NC, Number of Cycles; CP1, First Cycle Period; CP, Cycle Period; FF, Fundamental Frequency. Results refer to Kruskal-Wallis test with Dunn's multiple comparisons (*P*-value).

Table S2. Percentage and cumulative percentage of the first two axes of principal component analysis (PCA) with PCA loadings for the two axes extracted from the three acoustic properties

	PC1	PC2
Percentage	48.21	47.82
Cumulative percentage	48.21	96.03
Sound duration	-0.453	0.869
First cycle period	0.532	0.823
Fundamental frequency	-0.979	0.045

Table S3. Percentage and cumulative percentage of the first two axes of discriminant function analysis (DFA) with DFA loadings for the two axes extracted from the three acoustic properties

	DF1	DF2
Percentage	77.83	19.19
Cumulative percentage	77.83	97.02
Sound duration	-0.272	-0.491
First cycle period	-0.080	-0.680
Fundamental frequency	-0.925	0.176

Table S4. Morphological measurements of specimens, anterior sac of the swimbladder and sonic muscle. Values are means ± SD.

		**Swimbladder (cranial sac)**			**Sonic muscle**			
**Species (N)**	**SL (mm)**	**SBL (mm)**	**rSBL**	**SBH (mm)**	**SML (mm)**	**rSML**	**SMH (mm)**	**rSMH**
Serrasalmus elongatus (3)	91 ± 28	18.0 ± 7.0	0.20 ± 0.02	9.5 ± 3.0	8.1 ± 3.6	0.45 ± 0.02	7.7 ± 3.0	0.80 ± 0.09
Serrasalmus marginatus (3)	58 ± 1	10.7 ± 0.9	0.19 ± 0.01	8.0 ± 0.3	5.2 ± 0.3	0.49 ± 0.05	5.4 ± 0.7	0.67 ± 0.06
Serrasalmus compressus (1)	70	13.4	0.19	9.1	5.9	0.44	6.7	0.74
Serrasalmus rhombeus (3)	109 ± 8	23.5 ± 3.9	0.22 ± 0.02	14.2 ± 1.0	9.1 ± 1.0	0.39 ± 0.03	11.3 ± 1.2	0.79 ± 0.05
Serrasalmus eigenmanni (3)	103 ± 2	19.3 ± 1.7	0.19 ± 0.01	13.9 ± 0.7	6.7 ± 0.4	0.35 ± 0.03	9.8 ± 0.3	0.71 ± 0.03
Pygocentrus nattereri (2)	121 ± 10	27.0 ± 2.8	0.22 ± 0.01	15.2 ± 1.1	9.9 ± 1.2	0.37 ± 0.01	10.5 ± 0.6	0.69 ± 0.09

N, number of dissected individuals per species; SL, standard length
SBL, maximum swimbladder length; rSBL, relative swimbladder length (rSBL = SBL/SL); SBH, maximum swimbladder height; SML, maximum sonic muscle length; rSML, relative sonic muscle length (rSML = SML/SBL); SMH, maximum sonic muscle height: rSMH, relative sonic muscle height (rSMH = SMH/SBH).

Table S5. Results of the linear regressions of fish size against the two supplementary acoustic variables.

	Number of cycles			Cycle period (ms)		
	Equation	R^2	*P*-value	Equation	R^2	*P*-value
S. elongatus	$y = -0.166x + 31.69$	0.895	**0.0001**	$y = 0.0155x + 4.684$	0.542	**0.024**
S. marginatus	$y = 0.064x + 6.375$	0.780	**0.020**	$y = 0.001x + 6.775$	0.0006	0.963
S. compressus	$y = -0.0034x + 10.23$	0.001	0.946	$y = 0.034x + 4.075$	0.366	0.150
S. manueli	$y = 0.168x – 12.70$	0.425	0.348	$y = -0.084x + 19.05$	0.784	0.115
S. spilopleura	$y = -0.0875x + 17.08$	0.099	0.492	$y = 0.002x + 6.073$	0.0009	0.949
S. rhombeus	$y = -0.0106x + 8.413$	0.239	0.219	$y = 0.0228x + 5.117$	0.922	**0.0002**
S. eigenmanni	$y = 0.0073x + 7.576$	0.022	0.680	$y = 0.0239x + 5.097$	0.405	**0.048**
P. nattereri	$y = 0.0536x + 7.562$	0.647	**0.0002**	$y = 0.032x + 4.765$	0.967	**< 0.0001**

R^2, coefficient of determination; *P*-value in bold are significant.

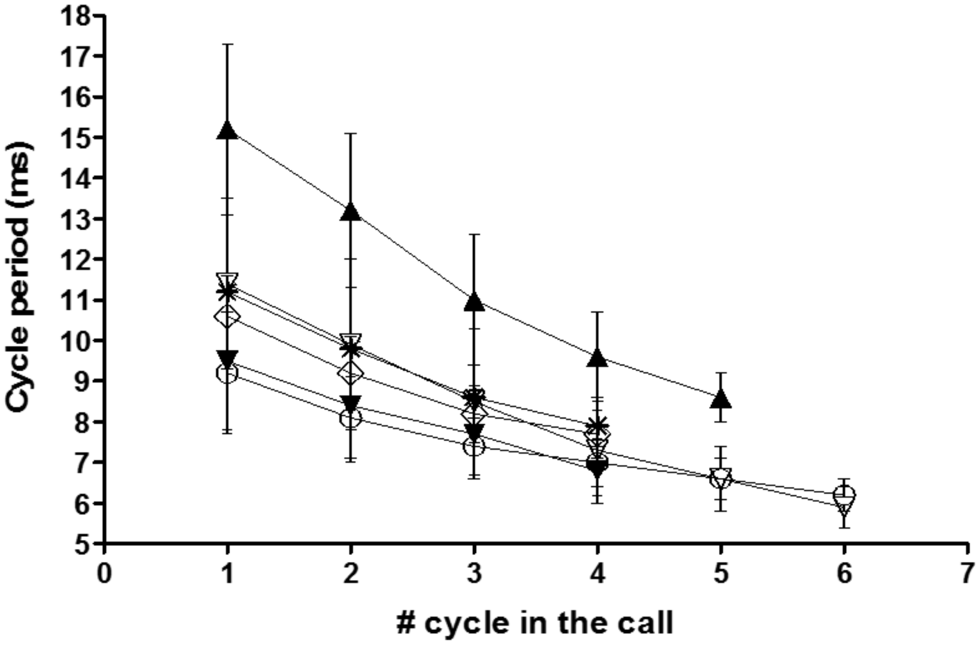

Figure S1. Means (± S.D.) of the successive cycle periods in the beginning of the calls in *Serrasalmus elongatus* (▽), *Serrasalmus manueli* (▲), *Serrasalmus spilopleura* (○), *Serrasalmus rhombeus* (◇), *Serrasalmus eigenmanni* (✳) and *Pygocentrus nattereri* (▼).

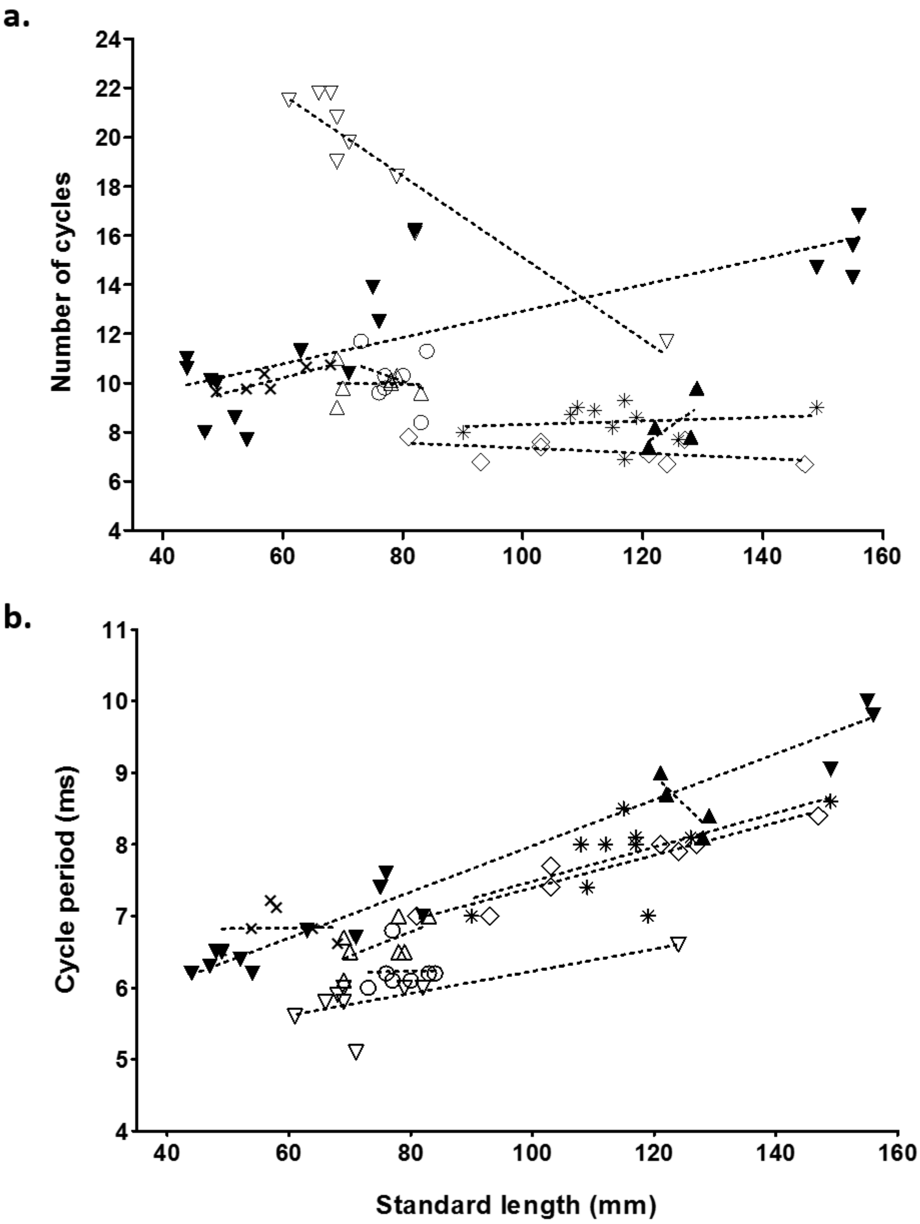

Figure S2. Influence of fish size on **(a)** number of cycles, **(b)** cycle period in 8 piranha species.
Note that some data concerning *P. nattereri* have been added from Millot and Parmentier (2014)[48]. Legend: *Serrasalmus elongatus* ▽, *Serrasalmus marginatus* ×, *Serrasalmus compressus* △, *Serrasalmus manueli* ▲, *Serrasalmus spilopleura* ○, *Serrasalmus rhombeus* ◇, *Serrasalmus eigenmanni* ✳ and *Pygocentrus nattereri* ▼.
